# Broad SARS-CoV-2 neutralization by monoclonal and bispecific antibodies derived from a Gamma-infected individual

**DOI:** 10.1016/j.isci.2023.108009

**Published:** 2023-09-22

**Authors:** Denise Guerra, Tim Beaumont, Laura Radić, Gius Kerster, Karlijn van der Straten, Meng Yuan, Jonathan L. Torres, Wen-Hsin Lee, Hejun Liu, Meliawati Poniman, Ilja Bontjer, Judith A. Burger, Mathieu Claireaux, Tom G. Caniels, Jonne L. Snitselaar, Tom P.L. Bijl, Sabine Kruijer, Gabriel Ozorowski, David Gideonse, Kwinten Sliepen, Andrew B. Ward, Dirk Eggink, Godelieve J. de Bree, Ian A. Wilson, Rogier W. Sanders, Marit J. van Gils

**Affiliations:** 1 Amsterdam UMC, location University of Amsterdam, Department of Medical Microbiology and Infection Prevention, Laboratory of Experimental Virology, Meibergdreef 9, Amsterdam 1105 AZ, the Netherlands; 2 Amsterdam Institute for Infection and Immunity, Infectious Diseases, Amsterdam, the Netherlands; 3 Amsterdam UMC, location University of Amsterdam, Department of Internal Medicine, Meibergdreef 9, Amsterdam 1105 AZ, the Netherlands; 4 Department of Integrative Structural and Computational Biology, The Scripps Research Institute, La Jolla, CA 92037, USA; 5 Center for Infectious Disease Control, National Institute for Public Health and the Environment (RIVM), 3721 MA Bilthoven, the Netherlands; 6 The Skaggs Institute for Chemical Biology, The Scripps Research Institute, La Jolla, CA 92037, USA; 7 Department of Microbiology and Immunology, Weill Medical College of Cornell University, New York, NY, USA

**Keywords:** Immunology, Virology, Structural biology

## Abstract

The pandemic caused by severe acute respiratory syndrome coronavirus 2 (SARS-CoV-2) has remained a medical threat due to the evolution of multiple variants that acquire resistance to vaccines and prior infection. Therefore, it is imperative to discover monoclonal antibodies (mAbs) that neutralize a broad range of SARS-CoV-2 variants. A stabilized spike glycoprotein was used to enrich antigen-specific B cells from an individual with a primary Gamma variant infection. Five mAbs selected from those B cells showed considerable neutralizing potency against multiple variants, with COVA309-35 being the most potent against the autologous virus, as well as Omicron BA.1 and BA.2, and COVA309-22 having binding and neutralization activity against Omicron BA.4/5, BQ.1.1, and XBB.1. When combining the COVA309 mAbs as cocktails or bispecific antibodies, the breadth and potency were improved. In addition, the mechanism of cross-neutralization of the COVA309 mAbs was elucidated by structural analysis. Altogether these data indicate that a Gamma-infected individual can develop broadly neutralizing antibodies.

## Highlights

• Primary infection with the SARS-CoV-2 Gamma variant elicits potent and broad antibodies
• COVA309 antibodies bind and neutralize a wide range of SARS-CoV-2 variants
• Bispecific COVA antibodies show increased neutralization breadth

## Graphical abstract

**Figure:**
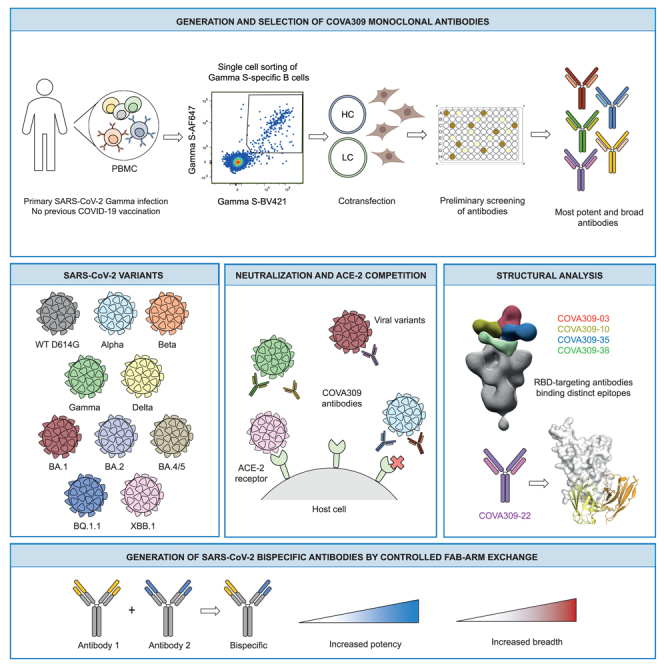

## Introduction

More than three years since the beginning of the coronavirus disease 2019 (COVID-19) pandemic, over 767 million infections and 6 million deaths caused by severe acute respiratory syndrome coronavirus 2 (SARS-CoV-2) have been reported to the World Health Organization (WHO), resulting in massive effects on global health and society in general.^1^ Fortunately, the accelerated development of multiple, safe, and effective COVID-19 vaccines has contributed to prevention of severe disease, reduced viral spread, and reduced stress on healthcare systems.^2^ Although vaccines remain the best approach to mitigate COVID-19, alternatives are needed for individuals at risk, such as the elderly and the immune-compromised, who may not develop an immune response strong enough to forestall serious disease outcomes.

One promising alternative is the prophylactic and therapeutic use of neutralizing monoclonal antibodies (mAbs). Multiple SARS-CoV-2-directed mAb products have been developed for the emergency treatment of mild-to-moderate COVID-19 cases. These comprise cocktail therapies, including casirivimab (REGN10933) in combination with imdevimab (REGN10987) by Regeneron Pharmaceutical, bamlanivimab (LY-CoV555) plus etesevimab (LY-CoV016) by Eli Lilly Company, tixagevimab (COV2-2196) plus cilgavimab (COV2-2130) by AstraZeneca, and two monotherapies, sotrovimab (VIR-7831) and bebtelovimab (LY-CoV1404/LY3853113), developed by Vir Biotechnology with GlaxoSmithKline and Eli Lilly, respectively.^3^ The target of these therapeutics is the homotrimeric spike (S) glycoprotein exposed on the virion surface, which mediates the fusion of viral and host membranes after attachment to the human angiotensin converting enzyme 2 (ACE-2) receptor.^4^^,^^5^ On the S protein, the major target of the neutralizing response is the receptor binding domain (RBD), which transiently shifts between up and down conformations, thus representing a target where some epitopes are differentially exposed depending on RBD state.^4^ Other regions of the S protein that are recognized by antibodies are the N-terminal domain (NTD) of the membrane-distal S1 head domain, and the conserved membrane-proximal S2 subunit that houses the fusion machinery and is generally targeted by more broadly reactive mAbs.^6^^,^^7^^,^^8^^,^^9^

More recently, the evolution of the wild-type (WT) virus into multiple variants that have acquired resistance to vaccines and mAb therapies has raised concerns about the longevity and efficacy of current treatment options, which are based on the original Wuhan Hu-1 S sequence. Some of these variants are characterized by higher transmissibility, virulence, and/or immune evasion compared to the WT virus, resulting in the WHO declaring the Alpha (Pango nomenclature B.1.1.7), Beta (B.1.351), Gamma (P.1), Delta (B.1.617.2), and Omicron (B.1.1.529) lineages as variants of concern (VOCs).^10^ In addition, variants of interest (VOIs) have also been reported worldwide. While the Alpha, Beta, Gamma, and Delta variants have a moderate number of mutations in the S protein, the latest Omicron variant, with its sub-lineages, contains more than 30 amino acid changes, of which almost half are located in the RBD, underpinning its substantial shift in antigenicity compared to early variants.^11^^,^^12^ Several studies have indeed reported that the antigenic drift in the Omicron sub-lineages has resulted in a 20- to 40-fold reduction in the neutralization potency of sera from vaccinated and previously infected individuals.^13^^,^^14^^,^^15^^,^^16^ In addition, a substantial or complete loss in neutralizing activity has also been observed for most commercial therapeutic mAbs, with sotrovimab, bebtelovimab, and the combination of tixagevimab with cilgavimab being the only antibodies still showing activity against Omicron BA.1, albeit with a 5- to 100-fold loss of potency. Moreover, sotrovimab and tixagevimab plus cilgavimab have been shown to lose efficacy against Omicron BA.2, and the BQ.1.1 and XBB.1 lineages are resistant to bebtelovimab.^14^^,^^15^^,^^16^^,^^17^^,^^18^^,^^19^^,^^20^^,^^21^ In addition, the emergence of resistance-associated mutations after sotrovimab administration has been recently reported.^22^^,^^23^

The marked reduction in VOCs neutralization by the current mAbs and vaccine-induced sera highlights the importance and the need for the discovery of novel broadly reactive neutralizing mAbs covering different SARS-CoV-2 strains and potential future emerging variants. In addition, although the isolation of mAbs from vaccinated and convalescent patients infected with the WT SARS-CoV-2 virus has been reported,^6^^,^^24^^,^^25^ reports describing the discovery of such antibodies from VOC-infected individuals are limited.^26^ Here, we describe the isolation of a set of human mAbs isolated from a convalescent unvaccinated COVID-19 patient who experienced a confirmed primary infection with the Gamma variant. The antibodies display a variety of functionalities against the autologous virus but also against heterologous VOCs, including WT, Alpha, Beta, Delta, Omicron BA.1, BA.2, BA.4/5, BQ.1.1, and XBB.1. The specificities were corroborated by structural analyses. In particular, COVA309-35 had very potent neutralizing activity against Omicron BA.1 and BA.2 but less against Delta, while COVA309-22 retained binding and neutralization, although to a lesser extent, against more recent Omicron sub-lineages. Combining COVA309-35 with COVA1-18 and COVA1-16, previously isolated from a Wuhan Hu-1-infected individual,^6^ yielded bispecific antibodies (bsAbs) with increased breadth and potency.

## Results

### Selection of SARS-CoV-2 spike-specific B cells and antibodies from a Gamma-infected individual

To study the B cell response induced by infection with the SARS-CoV-2 Gamma variant and isolate mAbs with novel specificities, we selected samples from an unvaccinated 32-year-old female, COSCA309, who experienced upper respiratory tract infection but did not require hospital admission, following a primary sequence-confirmed Gamma infection. COSCA309 was enrolled in the COSCA study (NL 73281.018.20) that was set up to facilitate investigation of VOC-specific B cell responses and isolation of broad and potent neutralizing mAbs against SARS-CoV-2 and its variants.^6^^,^^11^

**Figure 1:**
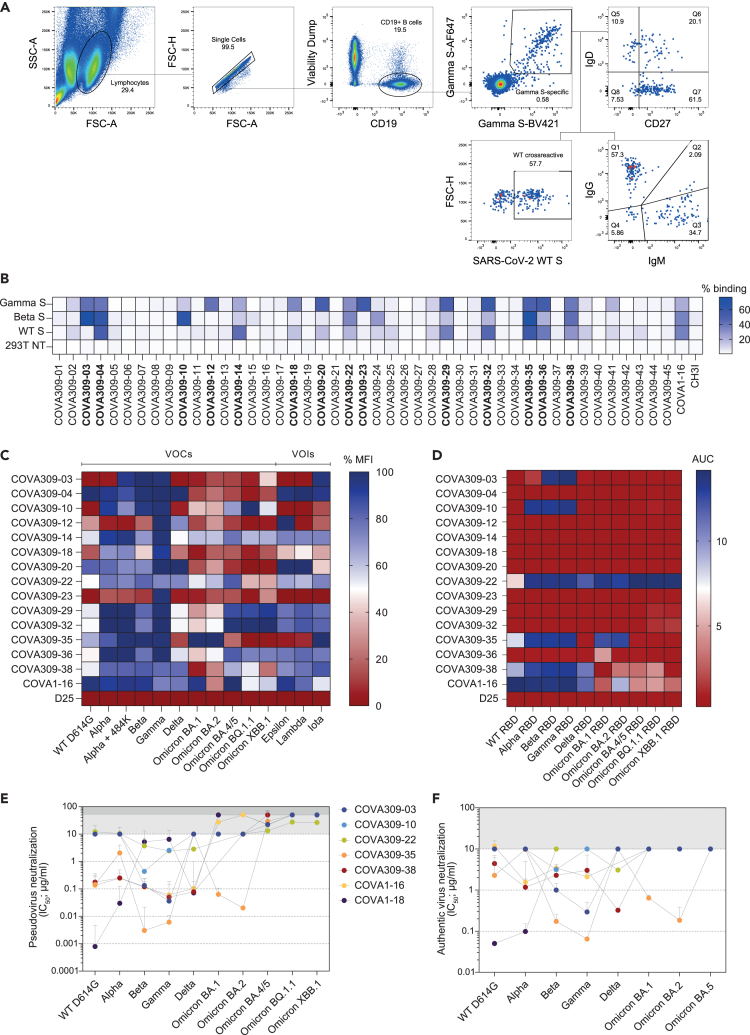
Selection of B cells and antibodies from a SARS-CoV-2 Gamma-infected individual (A) Sorting strategy of singlet viable CD19^+^ SARS-CoV-2 Gamma S-specific B cells. Gamma S-specific B cells were selected by double staining of Gamma S labeled with two different fluorescent dyes (Gamma S-AF647, Gamma S-BV421). In addition, Gamma S-positive B cells were stained for IgD, CD27, IgG, and IgM expression. Frequency of WT S cross-reactive B cells is also indicated. SSC-A, side scatter area; FSC-H, forward scatter height; FSC-A, forward scatter area. (B) Flow cytometry-based screening of HEK293T-produced non-purified COVA309 supernatants against Gamma, Beta, and WT S expressed on HEK293T cells, shown in the percentage of binding. 293T NT, non-transfected cells. COVA1-16 and CH3I antibodies were included as positive and negative controls, respectively. 14 mAbs (in bold) were selected for larger scale expression. (C) Heatmap showing the percentage of mean fluorescence intensity (MFI) of HEK293F-produced and purified COVA309 mAbs binding to SARS-CoV-2 variant S expressed on HEK293T cells, as assessed by flow cytometry. VOCs, variants of concern; VOIs, variants of interest. COVA1-16 and D25 (an RSV F specific mAb) are used as positive and negative controls, respectively. (D) Heatmap depicting the binding of COVA309 mAbs to SARS-CoV-2 variant RBDs, as determined by ELISA. Color scale indicates the area under the curve (AUC) for each mAb. (E) Half maximal inhibitory concentrations (IC_50_) of SARS-CoV-2 VOC pseudoviruses neutralization for COVA309 mAbs, COVA1-16, and COVA1-18.^6^^,^^39^^,^^40^ The cutoff was set at 10 μg/mL (light gray bar) for variants up to Omicron BA.2, and at 50 μg/mL (dark gray bar) for Omicron BA.4/5, BQ.1.1, and XBB.1. Each dot represents the mean value from two or three experiments in which three or four replicates per mAb were performed. (F) Neutralization of authentic SARS-CoV-2 viruses by COVA309 mAbs. The cutoff was set at 10 μg/mL (gray bar). Color code is the same as for the pseudovirus neutralization. COVA1-16 and COVA1-18 mAbs were tested against WT, Alpha, Beta, Gamma, Omicron BA.1, BA.2, and BA.5 to corroborate the pseudovirus neutralization findings. Each dot represents the mean value from one or two experiments performed in duplo.

We collected blood samples from COSCA309 approximately 40 days after symptom onset. After blood sample collection, peripheral blood mononuclear cells (PBMCs) were isolated and Gamma S-specific B cells were selected by flow cytometry-based single cell sorting using a stabilized Gamma S probe^6^ in two colors, in addition to the WT S protein in a third color. The flow cytometry analysis of the donor PBMCs showed a frequency of 0.58% Gamma S-specific B cells (Gamma S-AF647^+^, Gamma S-BV421^+^) among the total pool of B cells (CD19^+^Via^−^), which were shown to be predominantly memory B cells (CD27^+^IgD^−^, 61.5%) (Figure 1A). Within these Gamma S-specific B cells, the majority expressed immunoglobulin G (IgG^+^) (57.3%), although a considerable portion of the S-specific B cells were IgM^+^ (34.7%). Co-staining with WT SARS-CoV-2 S protein indicated that 57.7% of Gamma S-specific B cells cross-reacted with the WT strain (Figure 1A). After gene amplification and single-cell cloning, we obtained a total of 45 productive IgG heavy and light chain (HC/LC) pairs. The genetic signatures of the Gamma S-specific B cells were compared to the International Immunogenetics Information System (IMGT) germline repertoire^27^ (Table S1). An overall increase in the usage of certain genes, such as IGHV3-53/3–66, IGHV1-2, IGHV3-30, and IGHV1-69, has been previously reported in COVID-19 patients infected with the WT SARS-CoV-2 virus.^6^^,^^28^^,^^29^^,^^30^^,^^31^^,^^32^^,^^33^^,^^34^^,^^35^ Strikingly, none of the mAbs isolated in this study has been found to use the most dominant IGHV3-53 gene segment (Table S1), whereas IGHV1-69 was found to be frequently used by the sorted Gamma S-specific B cells (15.6%), followed by IGHV3-7 and IGHV3-23 (11.1% each) and IGHV4-59 (8.9%) (Figure S1A). The median somatic hypermutations (SHM) was 2%, in line with SHM levels observed after infection with the WT SARS-CoV-2.^6^^,^^25^^,^^36^^,^^37^ We did not find any substantial difference in the heavy complementary determining region 3 (CDRH3) length of isolated Gamma S-specific B cells (mean 16 amino acids) compared to the average length of 15 amino acids generally present in the human naive repertoire.^38^ Following expression of the 45 mAbs in human embryonic kidney (HEK) 293T cells and subsequent screening of the supernatants by a flow cytometry-based binding assay against the autologous Gamma S, as well as against Beta and WT S, a total of 14 mAbs were selected based on binding potency and breadth for large-scale protein production and purification (Figure 1B).

We first assessed COVA309 mAbs binding specificities. Using flow cytometry, we tested the 14 antibodies for binding to HEK293T-transfected cells expressing a large panel of full-length membrane-expressed S, including WT D614G, VOCs (Alpha, Alpha with E484K, Beta, Gamma, Delta, Omicron BA.1, BA.2, BA.4/5, BQ.1.1, and XBB.1), and VOIs (Epsilon, Lambda, and Iota) (Figure 1C). Besides binding to the autologous Gamma S protein, 11 out of the 14 mAbs also recognized the Beta variant, in line with the presence of high similarity between the S mutations in these strains. In general, COVA309 mAbs showed reduced binding to the WT D614G S compared to Beta and Gamma variants, while most of them still recognized the Alpha S. Even though some of the mAbs showed decreased binding to Delta and the Omicron BA.1 and BA.2 variants, almost half of the tested mAbs still recognized the S of Omicron BA.4/5, BQ.1.1, and XBB.1. This may indicate that approximately 30%–40% of the sorted Gamma S-specific B cells were likely to be cross-reactive to more recent Omicron sub-variants. Furthermore, COVA309-35 was unique since it showed the strongest binding to the Omicron BA.1 and BA.2 sub-lineages. In total, 7 mAbs recognized Epsilon, Lambda, and Iota VOIs. In addition to the flow cytometry binding data to full-length S, we performed an enzyme-linked immunosorbent assay (ELISA) with soluble RBD proteins of the WT, Alpha, Beta, Gamma, Delta, Omicron BA.1, BA.2, BA.4/5, BQ.1.1, and XBB.1 variants and showed that 5 COVA309 mAbs are directed toward the RBD, while the others probably target other epitopes on the S, such as the NTD and the S2 subunit (Figure 1D). Among the RBD-targeting mAbs, COVA309-22, -35, and -38 were the broadest, with COVA309-22 binding to all RBDs of the tested variants, while COVA309-03 and -10 seemed to be more strain specific. Overall, these data indicate that following a primary infection with the Gamma variant, broadly reactive mAbs against other viral strains can be elicited.

### Gamma-elicited antibodies show potent and broad neutralization

In addition to the binding data, neutralizing activity of the 14 selected mAbs was assessed in a lentiviral-based pseudovirus neutralization assay against the autologous Gamma strain (Figure S1B). The RBD-targeting COVA309-03, -35, and -38 mAbs exhibited considerable neutralizing potency against the autologous Gamma variant, with half maximal inhibitory concentrations (IC_50_) ranging from 0.007 to 0.067 μg/mL (Figure 1E). Among them, COVA309-35 was the most potent (IC_50_ 0.007 μg/mL). In addition, two other mAbs (COVA309-10 and −22) were able to neutralize the autologous strain (IC_50_ 2.6 μg/mL and 2.4 μg/mL, respectively) (Figure 1E). Since we aimed at finding cross-neutralizing mAbs, we tested the five COVA309 mAbs for broad neutralization against heterologous SARS-CoV-2 lineages, including the WT D614G, Alpha, Beta, Delta, Omicron BA.1, BA.2, BA.4/5, BQ.1.1, and XBB.1 strains (Figure 1E). Compared to autologous Gamma neutralization, we observed similar potencies against Beta, suggesting a primary role of key amino acid mutations shared between these two lineages, mainly located in the RBD (K417 N/T, E484K, and N501Y). Overall, most COVA309 mAbs did not show neutralization below the cutoff of 10 μg/mL against the Omicron BA.1 and BA.2 or below the cutoff of 50 μg/mL against the Omicron BA.4/5, BQ.1.1, and XBB.1 sub-lineages. However, COVA309-35 still retained potent neutralization activity against Omicron BA.1 and BA.2 variants (IC_50_ 0.07 μg/mL and 0.02 μg/mL, respectively), while it was less effective against Alpha (IC_50_ 2.3 μg/mL) and did not neutralize the Delta strain with IC_50_ < 10 μg/mL. On the contrary, antibody COVA309-38 neutralized the Delta variant (IC_50_ 0.08 μg/mL), but not Omicron BA.1 and BA.2. When tested against the latest Omicron sub-variants, COVA309-22 maintained neutralization activity (IC_50_ 13.6, 27.4, and 26.7 μg/mL against Omicron BA.4/5, BQ.1.1, and XBB.1, respectively), in accordance with the observed binding breadth (Figures 1C and 1D).

Next, we assessed the ability of the five COVA309 mAbs to neutralize authentic SARS-CoV-2 viruses, including WT, Alpha, Beta, Gamma, Delta, Omicron BA.1, BA.2, and BA.5 strains (Figures 1F and S1C). COVA309-03 neutralized primary Beta and Gamma viruses with an IC_50_ of 1.03 and 0.3 μg/mL, respectively, whereas COVA309-10 only neutralized Beta (IC_50_ 3.2 μg/mL). COVA309-35 and -38 were still highly effective in neutralizing multiple primary viruses. COVA309-35 neutralized autologous Gamma virus efficiently (IC_50_ 0.07 μg/mL) but showed a decrease in neutralization against Alpha and Delta viruses (IC_50_ 9.7 μg/mL and >10 μg/mL, respectively), while maintaining neutralization of Omicron BA.1 and BA.2 variants (IC_50_ 0.6 and 0.2 μg/mL, respectively). COVA309-38 neutralized primary variants with IC_50_ ranging from 0.3 μg/mL to 4.7 μg/mL but was not effective against the Omicron BA.1 and BA.2 strains (IC_50_ > 10 μg/mL). None of the tested mAbs showed neutralization against the primary Omicron BA.5 virus with IC_50_ values below the cutoff of 10 μg/mL. Compared to the pseudovirus neutralization, the neutralization potency against authentic SARS-CoV-2 strains was generally somewhat reduced, consistent with previous studies,^6^^,^^41^ but the five neutralizing mAbs were overall similar between primary virus and pseudovirus neutralization assays in terms of activity and patterns of neutralization, which is confirmed by a strong correlation between the two assays (Figure S1D, Spearman correlation, r = 0.8460, p < 0.0001) and is also in line with previous reports.^41^^,^^42^

When we examined the genetic signatures of the five neutralizing versus non-neutralizing COVA309 mAbs, we found that COVA309-03, -10, -22, and -38 used genes belonging to the IGHV3 family (IGHV3-48, IGHV3-23, IGHV3-33, and IGHV3-7, respectively), while COVA309-35 was encoded by the dominant IGHV1-69 gene. The five mAbs used diverse IGKV/IGLV genes (IGKV1-5 for COVA309-03, IGKV1-39 for COVA309-10 and −22, IGKV1-27 for COVA309-35, and IGLV6-57 for COVA309-38). The neutralizing mAbs had low levels of SHM (median 1.7%) and average heavy and light CDR3 lengths (10–23 amino acids and 9–10, respectively). These properties were similar to those of the Gamma-elicited non-neutralizing mAbs and previously described RBD-targeting mAbs that also present low SHM. However, the genetic features of mAbs isolated after WT infection, which predominantly use gene segments IGHV3-53/3–66 and IGHV3-30,^6^^,^^36^^,^^38^ were found to differ from the gene usage of Gamma-elicited mAbs (Figure S1A; Table S1). Together these findings indicate that, despite certain features being preserved between WT- and VOC-induced mAbs, other signatures seem to be strain specific.

### Structural analysis of COVA309 mAbs explains their ability to cross-neutralize

**Figure 2:**
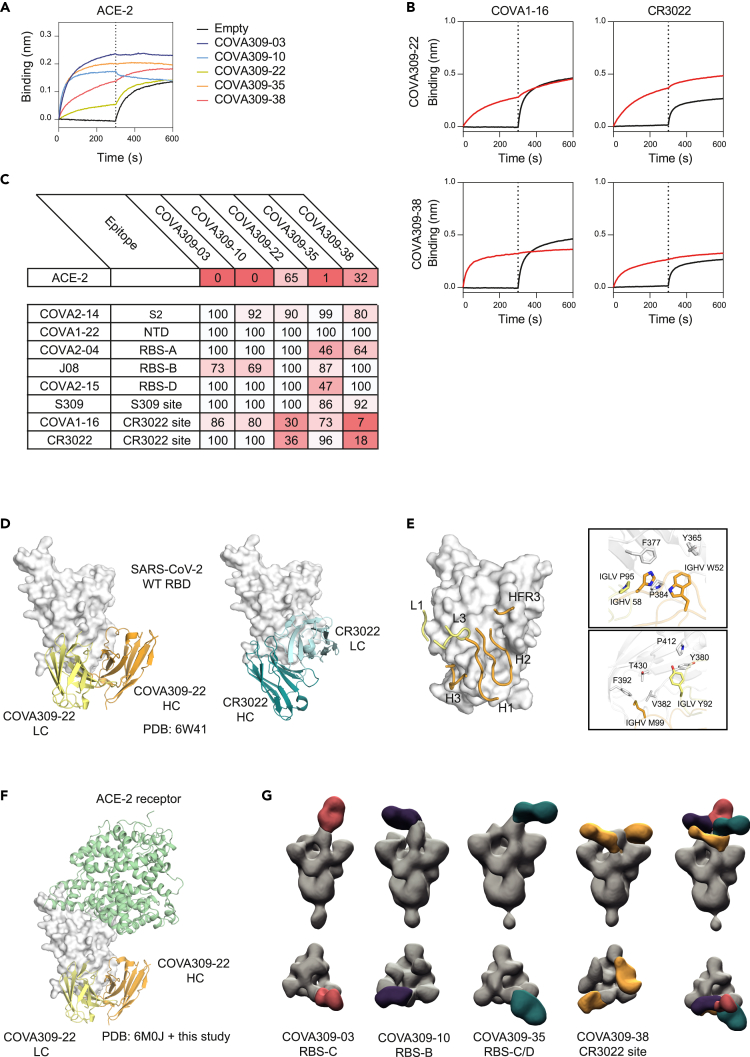
COVA309 mAb epitope determination (A) Biolayer interferometry plot depicts competition between the COVA309 mAbs and ACE-2, for binding to Gamma S coated on the chip. The black curves represent the baseline. (B) Example of BLI plots showing COVA309-22 and -38 mAbs competing with COVA1-16 and CR3022 for binding to the WT S. The black curves represent the baseline, when no analyte was added. (C) Heatmap showing the percentage of residual binding of the ACE-2 receptor and other known SARS-CoV-2 mAbs after competition with COVA309 mAbs, as determined by BLI assay. For the mAbs competition, WT S was loaded on biosensors, and COVA2-14 (S2 binder), COVA1-22 (NTD binder), COVA2-04 (RBS-A), J08 (RBS-B), COVA2-15 (RBS-D), S309 (S309 site), COVA1-16 (CR3022 site), and CR3022 (CR3022 site)^6^^,^^45^^,^^46^^,^^47^ were included as competitors. (D) Crystal structure of COVA309-22 Fab in complex with SARS-CoV-2 WT RBD at a 3.7 Å resolution (left). The HC and LC are colored in orange and yellow, respectively. CR3022 Fab (right) is reported as a comparison (PDB: 6W41). (E) Detailed representation of the main residues involved in the COVA309-22-WT RBD interaction. (F) COVA309-22 HC (orange) and LC (yellow) bind the base and lateral face of the RBD, far from the ACE-2 receptor binding site (green; PDB: 6M0J). (G) Front view (top row) and top view (bottom row) of low-resolution NS-EM reconstructions of COVA309-03, -10, -35, and -38 in complex with either Omicron 6P S or Gamma 6P S. Composite NS-EM maps of COVA309 Fabs indicate that all COVA309 mAbs align on one RBD in the up conformation.

To study the mechanism of binding of the neutralizing COVA309 mAbs, we performed a biolayer interferometry (BLI) assay measuring competition between COVA309 mAbs and recombinant soluble human ACE-2 receptor for binding to autologous Gamma S (Figure 2A). COVA309-03, -10, and -35 were able to block ACE-2 binding to Gamma S, suggesting that they target an epitope in close vicinity to the ACE-2-binding site on the apical area of the RBD. Alternatively, depending on the angle of approach to the RBD, they may cause steric hindrance with the receptor, therefore precluding ACE-2 interaction with S. These data are consistent with observations that many potent RBD-specific mAbs neutralize the virus by binding to a region that mediates ACE-2 receptor engagement, or by inhibiting the receptor attachment through specific approaching angles.^43^^,^^44^ On the contrary, mAbs COVA309-22 and -38 were unable to block ACE-2 binding, suggesting that they might have different binding sites and mechanisms of action than ACE-2 blocking (Figures 2A and 2C).

Next, to acquire more insight into the binding site specificities of the COVA309 mAbs, we tested their binding by BLI to the WT SARS-CoV-2 S coated on biosensors and determined cross-competition with previously characterized mAbs. The mAbs directed to the RBD can be classified based on their binding epitope, targeting either sub-sites in receptor binding site, (RBS)-A, -B, -C, -D, or the more conserved S309 and CR3022 sites.^45^^,^^46^ We included mAbs belonging to distinct classes, comprising COVA2-14 against S2, COVA1-22 against NTD, COVA2-04 against RBS-A, J08 against RBS-B, COVA2-15 against RBS-D, S309 against the S309 site, and COVA-16 and CR3022 against the CR3022 epitope^6^^,^^45^^,^^46^^,^^47^ (Figures 2B, 2C, and S2A). COVA309-22 and -38 strongly interfered with binding of COVA1-16 and CR3022, indicating that they target the CR3022 site on the lateral face of the RBD, which is highly conserved among variants (Figures 2B and 2C). COVA309-35 competed with COVA2-04 and COVA2-15 (46% and 47% residual binding, respectively), suggesting that its epitope partially overlaps with the regions targeted by the mentioned mAbs, or that the binding of COVA309-35 causes steric hindrance or induces a conformation change of the S which reduces the binding of COVA2-04 and COVA2-15. While we did not observe substantial competition of COVA309-03 with any of the tested mAbs, COVA309-10 showed a partial competition with RBS-B-targeting mAb J08.

We next determined the structural features and antigen recognition of the isolated COVA309 mAbs. We only obtained a crystal structure of the COVA309-22 antibody binding fragment (Fab) in complex with SARS-CoV-2 WT RBD at 3.7 Å resolution. In agreement with the BLI data, COVA309-22 interacts with a conserved RBD site that largely overlaps with that of CR3022 but in a different binding mode^46^ (Figure 2D). The HC and LC of COVA309-22 and CR3022 bind the RBD with a swapped orientation, suggesting that, although the footprint is the same, the residues involved in the antigen recognition and interaction are different.

The three CDRs of the COVA309-22 HC (CDRH1, H2, and H3) account for the majority of the interactions, burying 31 Å^2^, 377 Å^2^, and 232 Å^2^ of surface area (BSA), respectively, while the CDRL1, CDRL3, and HFR3 contribute to 73 Å^2^, 280 Å^2^, and 64 Å^2^ of the total 1057 Å^2^ BSA. Aromatic residues of COVA309-22 IGHV W52, H58, and IGLV P95 stack with RBD residues Y365, F377, and P384, whereas IGLV Y92 contacts RBD-Y380 and P412. Moreover, IGHV M99 forms hydrophobic interactions with RBD-V382, F392, and T430 (Figure 2E). In accordance with competition experiments, the structure confirmed that the ACE-2 receptor binding site is far from the epitope recognized by this antibody and that COVA309-22 does not interfere with receptor binding (Figure 2F). In parallel, we generated lower-resolution negative-stain electron microscopy (NS-EM) reconstructions of the other COVA309 mAbs. COVA309-03, -10, and -38 were analyzed in complex with 6P-stabilized Gamma S, while COVA309-35 was complexed with 6P-stabilized Omicron BA.1 S protein (Figures 2G and S2B). By using reference models from other mAbs, including CV07-270, CV07–250, C110, and DH1047,^48^^,^^49^ we could determine the epitope for each mAb (Figure S2C). In accordance with BLI competition data, COVA309-35 Fab in complex with the Omicron 6P S trimer showed that this antibody binds the apical part of the open, up-state RBD, targeting a region which encompasses the RBS-C and RBS-D regions^50^^,^^51^ (Figure 2G). This corroborates the competition of COVA309-35 with COVA2-15 that binds RBS-D. In addition, RBS-C harbors L452, mutated in the Delta strain, explaining why COVA309-35 does not neutralize Delta. This finding is consistent with the ACE-2 competition data and shows that COVA309-35 resembles the binding characteristics of mAb C110, which was also reported to target a similar epitope^52^ (Figure S2C). The NS-EM maps obtained from COVA309-03, -10, and -38 Fabs complexed with the Gamma 6P trimer demonstrated that all the antibodies could be aligned on the RBD in the up conformation, although COVA309-38 was also found to recognize the RBD in a partial down state. COVA309-10 binds RBS-B, on the apical part of the RBD, which includes amino acids at positions 478, 484, and 452, in accordance with our ACE-2 competition results and explains the loss in neutralizing activity against Delta and the Omicron sub-lineages that have different amino acids at these positions compared to Gamma. COVA309-03 targets RBS-C which also includes residues 484 and 452, thereby explaining the narrow neutralizing activity of this antibody. Lastly, NS-EM structures revealed that COVA309-38 binds the CR3022 site, in agreement with the competition to COVA1-16 and CR3022 itself (Figures 2B, 2C, and 2G).

### COVA309 mAbs contribute to potent and broad SARS-CoV-2 neutralization when incorporated into bsAbs

Next, we investigated whether antibodies with complementary SARS-CoV-2 neutralizing potencies could be combined in a bsAb format to achieve greater neutralization breadth. We focused on bsAbs that target distinct epitopes, including the most potent COVA309 mAbs, COVA309-35 and -38, and COVA1-18 and COVA1-16, two neutralizing mAbs isolated during the first wave of the pandemic.^6^^,^^39^^,^^40^ We have previously reported on their distinct ability to recognize SARS-CoV-2 variants and indicated how COVA1-18 retains potency against Delta, while COVA1-16 is broadly reactive but with limited potency against the Omicron strains (Figure 1E).^13^

**Figure 3:**
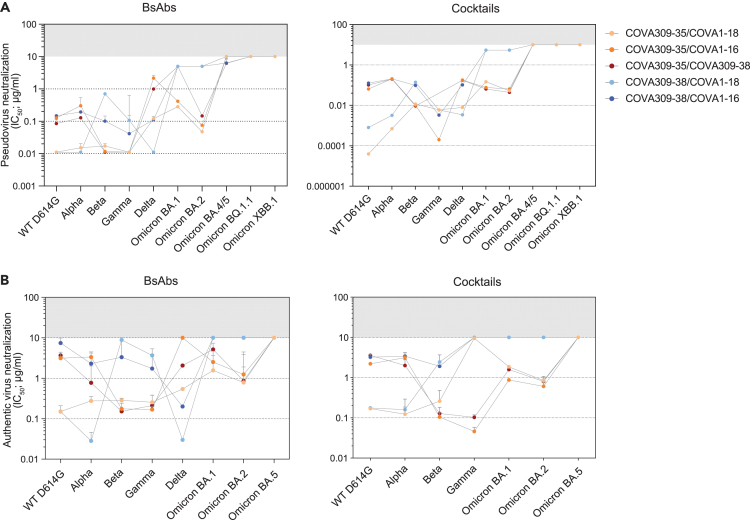
Characterization of bsAbs and antibody cocktails (A) IC_50_ values for pseudovirus neutralization of SARS-CoV-2 VOCs by bsAbs (left) and corresponding antibody cocktails (right). For antibody cocktails, a 1:1 ratio was used. The cutoff was set at 10 μg/mL (gray bar). Each dot represents the mean value from two or three experiments in which three or four replicates per mAb were performed. (B) Neutralization of authentic SARS-CoV-2 viruses by bsAbs and antibody cocktails. The cutoff was set at 10 μg/mL (gray bar). Each dot represents the mean value from one or two experiments performed in duplo.

We generated a total of five bsAbs by combining COVA309-35 and -38 together or in combination with either COVA1-18 or COVA1-16 and studied the neutralizing potency of the bsAbs and corresponding antibody cocktails against WT D614G, Alpha, Beta, Gamma, Delta, Omicron BA.1, BA.2, BA.4/5, BQ.1.1, and XBB.1 variants in a pseudovirus neutralization assay. Although COVA309-35 mAb alone remained the most potent antibody against the Beta and Gamma pseudoviruses (IC_50_ 0.006 and 0.007 μg/mL, respectively) (Figure 1E), its ability to cross-neutralize other VOCs was significantly improved when combined in the bsAb format with COVA1-18, COVA1-16, or COVA309-38, gaining potency against Delta (IC_50_ 0.1, 2.2, and 0.9 μg/mL, respectively) and retaining strong neutralizing activity against Omicron BA.1 and BA.2, with IC_50_ values ranging from 0.05 to 5.0 μg/mL (Figure 3A, left panel). Moreover, COVA309-35 together with COVA1-18, COVA1-16, and COVA309-38 neutralized WT D614G (IC_50_ 0.01, 0.1, and 0.09 μg/mL, respectively) and Alpha (IC_50_ 0.02, 0.3, and 0.1 μg/mL, respectively). When we tested bsAb COVA309-38 with COVA1-18 and COVA1-16, neutralization was improved compared to the parental COVA309-38 mAb (IC_50_ from 0.01 to 10 μg/mL), although activity against Omicron BA.1 and BA.2 was lower compared to bsAbs involving COVA309-35. COVA309-38 in combination with COVA1-16 and COVA309-35 showed neutralization of Omicron BA.4/5 (IC_50_ 6.5 and 6.3 μg/mL, respectively), while the other bsAbs did not neutralize Omicron BQ.1.1 and XBB.1 with IC_50_ values below the cutoff of 10 μg/mL. For some combinations, in particular COVA309-35 together with COVA309-38 against Omicron BA.1 and BA.2, the corresponding antibody cocktails appeared superior compared to the bsAb formats (0.06 versus 5.0 μg/mL and 0.04 versus 0.2 μg/mL for cocktails and bsAbs against Omicron BA.1 and BA.2, respectively), indicating that antibody avidity may be important for the neutralization potency and suggesting that both Fab regions are needed for efficient virus neutralization and clearance, as previously shown for other mAbs.^39^^,^^40^ Overall, these data indicate that when combined in multispecific formats, breadth of COVA309-35 and COVA309-38 is improved over the parental mAbs.

In addition to pseudovirus neutralization data, we also examined the neutralizing activity of the bsAbs against primary, authentic SARS-CoV-2 viruses, including WT, Alpha, Beta, Gamma, Delta, Omicron BA.1, BA.2, and BA.5 (Figure 3B, left panel). COVA309-35 in combination with either COVA1-18 or COVA309-38 appeared to have the highest potency and breadth, showing neutralization of all primary SARS-CoV-2 strains up to Omicron BA.1 and BA.2. COVA309-35 together with COVA1-16 also neutralized all variants except Delta and Omicron BA.5, with a slightly lower potency (IC_50_ from 0.2 to 3.4 μg/mL). In accordance with the lack of neutralization capacity of COVA309-38 against Omicron BA.1 and BA.2, we did not observe any activity of the bsAbs involving COVA309-38 against these strains. Moreover, similarly to the pseudovirus neutralization data, the bsAbs did not neutralize Omicron BA.5 with IC_50_ values < 10 μg/mL. Neutralization data of antibody cocktails against authentic SARS-CoV-2 viruses are also reported in Figure 3B, right panel.

## Discussion

SARS-CoV-2 has been shown to rapidly evolve into antigenically distinct variants, reducing the protective effect of natural infection as well as vaccination. The SARS-CoV-2 Omicron variants that outcompeted the Delta lineage indeed show higher resistance to sera from naturally infected and/or vaccinated individuals.^13^^,^^14^^,^^15^^,^^16^^,^^17^^,^^18^ Therefore, it is important to have potent therapeutics available, especially for the elderly and the immune-compromised. Different therapeutics have seen market introduction including several cocktails and mAbs. The mAbs, mAb cocktails, or bsAbs should ideally be able to prevent viral escape and be broad enough to recognize diverse future SARS-CoV-2 lineages. However, most commercially available mAb products, including sotrovimab and bebtelovimab, have been shown to lose potency against the latest Omicron sub variants.^15^^,^^17^^,^^18^^,^^19^^,^^20^^,^^21^ Since we mainly depend on memory B cells for protection against serious disease and future outbreaks, it is important to study the immune repertoire of convalescent individuals to understand the breath and potency of the memory B cell response. In this study, we were able to demonstrate that Omicron and Delta neutralizing mAbs derived from memory B cells were present in a Gamma-infected individual. This finding is in contrast with the relatively poor cross-reactive serum response observed for COSCA309,^11^ suggesting that the memory B cell response might be more versatile and therefore more adequately prepared to respond to future variants and be able to protect against severe disease.

The Gamma-elicited COVA309 mAbs differ substantially when comparing VOC binding, pseudoviru,s and primary virus neutralization. For example, COVA309-35 and -38 mAbs appear to have distinct S binding and neutralizing patterns against Omicron and Delta variants. Together, these mAbs can cover a broad range of antigenic variation within current and possible future SARS-CoV-2 strains. Therefore, we tested COVA309-35 and -38 together with COVA1-18 and COVA1-16 mAbs,^6^ either as bsAbs or as cocktails in a pseudovirus neutralization assay (Figure 3A). For bsAbs, lack of bivalent binding can be a disadvantage since the two different Fabs then should cooperate in binding to different epitopes on the same or adjacent S or S protomer. Although loss of bivalent binding can be observed for bsAbs compared to the combination therapy, the difference is small and, in general, both approaches provide broader antigenic coverage compared to the corresponding mAbs, especially the combination of COVA309-35 and COVA1-18. As a bsAb, this combination neutralizes all pseudovirus variants up to Omicron BA.2 with IC_50_ between 0.01 and 0.3 μg/mL, with the latter value being for BA.1, while as a cocktail, the range is from 0.001 to 0.2 μg/mL, again the least against BA.1.

In line with previous studies,^51^^,^^52^ the binding epitopes of the five neutralizing COVA309 mAbs are mainly located in the immunodominant RBD of the S (Figures 2C–2G). More specifically, we show that our SARS-CoV-2 mAbs target distinct RBD sites, within and outside the RBS. The differences in neutralization of the VOCs by the COVA309 mAbs could be explained by the structural analysis and binding studies. COVA309-35, which showed the highest potency against the autologous Gamma strain, as well as against Omicron BA.1 and BA.2, interferes with ACE-2 receptor binding and recognizes an epitope covering RBS-C and RBS-D when the RBD is in the up state, thereby being highly effective against the infectious S conformation. COVA309-03 was also found to bind RBS-C, but its binding specificity appears to be narrower as it neutralizes only the Beta and Gamma variants. This indicates that its binding strongly depends on 484K, which was confirmed by recognition of an Alpha strain that we engineered to contain the E484K mutation (Figure 1C).

In addition to mAbs targeting the RBS, COVA309-38 was found to target a more conserved cryptic site at the base and lateral face of the RBD. mAbs directed at this RBD region are generally broadly reactive but weakly neutralizing, although few exceptions exist like COVA1-16^6^^,^^39^ and ADI-62113.^53^ COVA1-16 exhibits a special angle of approach and is able to directly compete with the receptor binding through steric hindrance, thereby neutralizing SARS-CoV-2 much more potently. As confirmed by BLI experiments and NS-EM, we showed that COVA309-38, in addition to COVA1-16, competes with CR3022 and COVA309-22 (Figures 2B, 2C, and S2D). In addition, ACE-2 competition data revealed that, despite targeting the base and lateral face of the RBD, this antibody is still able to partially compete with the receptor binding to the S, thereby in part resembling the mechanism of action of COVA1-16. The crystal structure of COVA309-22 showed that the antibody targets the same epitope as COVA309-38, explaining the breadth of the antibody, albeit with a lower potency compared to mAbs targeting the RBS.^51^ The epitope of COVA309-22 also extensively overlaps with that of previously described mAbs, including CR3022^46^ and EY6A,^54^ indicating once again that this RBD region represents a key site of vulnerability on the S protein.

Here, we demonstrate that potent and broadly reactive Gamma-elicited antibodies can be generated and can still neutralize other variants, including the highly distant Omicron sub-lineages. Despite the lack of neutralization of all variants tested by a single COVA309 mAb, the overall humoral response of the COSCA309 patient, represented by the sum of the isolated antibodies targeting diverse viral epitopes, appears to be very broad and would be expected to confer a benefit in the protection against more recent SARS-CoV-2 lineages. We also show that, when tested in bsAb and cocktail combinations with other mAbs, all antibodies show enhanced neutralization breadth and potency, suggesting that combining antibodies with different RBD epitopes and mechanisms of action can provide a better resistance to viral mutants and therefore lead to more effective therapeutics. In addition, it is important to study how SARS-CoV-2 evolution shapes and alters the antibody response compared to the ancestral strain. In this way, we can increase our knowledge on the specific features and signatures of broadly reactive mAbs that cover diverse viral strains and may aid in development of next-generation vaccines and mAb therapeutics.

### Limitations of the study

Study limitations include the lack of high-resolution structures for COVA309-03, -10, -35, and -38 mAbs and the absence of *in vivo* therapeutic models.

## STAR★Methods

### Key resources table

**Table:** 

REAGENT or RESOURCE	SOURCE	IDENTIFIER
**Antibodies**		

COVA2-14	Brouwer et al.^6^	N/A
COVA1-22	Brouwer et al.^6^	N/A
COVA2-04	Brouwer et al.^6^	N/A
COVA2-15	Brouwer et al.^6^	N/A
COVA1-16	Brouwer et al.^6^	N/A
J08	Torres et al.^47^	RRID: AB_2802551
S309	Pinto et al.^45^	RRID: AB_2941328
CR3022	Yuan et al.^44^^,^^46^	RRID: AB_2895620
CD19-AF700 (HIB19)	BioLegend	Cat# 302225
CD20-PE-CF594 (2H7)	BD Biosciences	Cat# 562295
CD27-PE (L128)	BD Biosciences	Cat# 340425
IgM-BV605 (MHM-88)	BioLegend	Cat# 314524
IgG-PE-Cy7 (G18-145)	BD Biosciences	Cat# 561298
viability-eF780	eBiosciences	Cat# 65-0865-14
PE-conjugated goat F(ab)’2 anti-human IgG	Southern Biotech	Cat# 2042-09
Horseradish peroxidase–conjugated goat anti-human IgG	Jackson Immunoresearch	Cat# 109-035-006

**Bacterial and virus strains**		

Chemically competent DH5α *Escherichia coli*	Thermo Fisher Scientific	Cat#: 12879416
SARS-CoV-2 WT authentic virus	German isolate; GISAID ID EPI_ISL 406862	European Virus Archive Global #026V-03883
SARS-CoV-2 Alpha authentic virus	hCoV-19/Netherlands/NoordHolland_20432/2020	European Virus Archive Global #014V-04031
SARS-CoV-2 Beta authentic virus	hCoV-19/Netherlands/NoordHolland_10159/2021	European Virus Archive Global #014N-04147
SARS-CoV-2 Gamma authentic virus	hCoV-19/Netherlands/NoordHolland_10915/2021	European Virus Archive Global #014V-04089
SARS-CoV-2 Delta authentic virus	hCoV-19/Netherlands/NH-RIVM-27142/2021	European Virus Archive Global #014V-04483
SARS-CoV-2 Omicron BA.1 authentic virus	hCoV-19/Netherlands/NH-RIVM-72291/2021	European Virus Archive Global #014V-04429
SARS-CoV-2 Omicron BA.2 authentic virus	RIVM	N/A
SARS-CoV-2 Omicron BA.5 authentic virus	RIVM	N/A
SARS-CoV-2 Omicron BQ.1.1 authentic virus	This paper	N/A
SARS-CoV-2 Omicron XBB.1 authentic virus	This paper	N/A

**Biological samples**		

Human peripheral blood mononuclear cells	This study, convalescent SARS-CoV2 infected individual	N/A

**Chemicals, peptides, and recombinant proteins**		

FastDigest PstI	Thermo Fisher Scientific	Cat# FD0615
FastDigest BamHI	Thermo Fisher Scientific	Cat# FD0054
Fast Digest Green buffer 10x	Thermo Fisher Scientific	Cat# B72
Polyethylenimine Hydrochloride (PEI)max	PolySciences	Cat# 24765-1
20 U RNAse inhibitor	Invitrogen	Cat# AM2696
First strand SuperScript III system	Invitrogen	Cat# 18080-051
Dithiothreitol (DTT)	Invitrogen	Cat# D1532
Poly-L-Lysine	Sigma-Aldrich	Cat# P1399
3,3, 5,5′-tetramethylbenzidine	Thermo Fisher Scientific	Cat# 229280010
Casein buffer	Thermo Fisher Scientific	Cat# 37528
ExpiFectamine™ CHO Reagent	Thermo Fisher Scientific	Cat# A29129
Phosphate-buffered saline (PBS)	Thermo Fisher Scientific	Cat# 10010023
Tris	Sigma-Aldrich	Cat# T7943
2-mercaptoethylamine (2-MEA)	Sigma	Cat# M9768-25G
Penicillin	Sigma-Aldrich	Cat# P3032-10MI
Streptomycin	VWR	Cat# 382-EU-100G

**Critical commercial assays**		

QuikChange Site-Directed Mutagenesis Kit	New England Biolabs	Cat# E0554S
Gibson Assembly	Thermo Fisher Scientific	N/A
BIrA500 biotin-ligase reaction kit	Avidity	EC 6.3.4.15
CaptureSelect™ CH1-XL Affinity Matrix	Thermo Fisher Scientific	Cat# 194346205L
Bac-to-Bac system	Life Technologies	Cat# 10359016
Nano-Glo Luciferase Assay System	Promega	Cat# N1130

**Experimental models: Cell lines**		

HEK293F cells	Thermo Fisher Scientific	Cat# R79007
HEK293T cells	ATCC	Cat# CRL-11268
HEK-293T-hACE2	Schmidt et al.^55^	RRID:CVCL_A7UK
Vero E6 cells	American Type Culture Collection	Cat# CRL 1586TM
ExpiCHO cells	Thermo Fisher Scientific	Cat# A29127
Sf9 cells	ATCC	Cat# CRL-1711
High Five cells	Life Technologies	Cat# B85502

**Recombinant DNA**		

SARS-CoV-2 WT spike	GenBank	MN908947.3
SARS-CoV-2 Gamma spike	Described in Caniels et al.^41^	N/A
SARS-CoV-2 WT D614G RBD gene fragment	Integrated DNA Technologies	GenBank: MN908947.3 Mutations in this paper
SARS-CoV-2 Alpha RBD gene fragment	Integrated DNA Technologies	GenBank: MN908947.3 Mutations in this paper
SARS-CoV-2 Beta RBD gene fragment	Integrated DNA Technologies	GenBank: MN908947.3 Mutations in this paper
SARS-CoV-2 Gamma RBD gene fragment	Integrated DNA Technologies	GenBank: MN908947.3 Mutations in this paper
SARS-CoV-2 Delta RBD gene fragment	Integrated DNA Technologies	GenBank: MN908947.3 Mutations in this paper
SARS-CoV-2 Omicron BA.1 RBD gene fragment	Integrated DNA Technologies	GenBank: MN908947.3 Mutations in this paper
SARS-CoV-2 Omicron BA.2 RBD gene fragments	Integrated DNA Technologies	GenBank: MN908947.3 Mutations in this paper
SARS-CoV-2 Omicron BA.4/5 RBD gene fragments	Integrated DNA Technologies	GenBank: MN908947.3 Mutations in this paper
SARS-CoV-2 Omicron BQ.1.1 RBD gene fragments	Integrated DNA Technologies	GenBank: MN908947.3 Mutations in this paper
SARS-CoV-2 Omicron XBB.1 RBD gene fragments	Integrated DNA Technologies	GenBank: MN908947.3 Mutations in this paper
Human ACE-2 receptor gene fragment	Integrated DNA Technologies	Ensembl ENSG00000130234
SARS-CoV-2 WT D614G pseudovirus gene fragment	Described in Caniels et al.^41^	N/A
SARS-CoV-2 Alpha pseudovirus gene fragment	Described in Caniels et al.^41^	N/A
SARS-CoV-2 Beta pseudovirus gene fragment	Described in Caniels et al.^41^	N/A
SARS-CoV-2 Gamma pseudovirus gene fragment	Described in Caniels et al.^41^	N/A
SARS-CoV-2 Delta pseudovirus gene fragment	Described in van Gils et al.^13^	N/A
SARS-CoV-2 Omicron BA.1 pseudovirus gene fragment	Described in van Gils et al.^13^	N/A
SARS-CoV-2 Omicron BA.2 pseudovirus gene fragment	Integrated DNA Technologies	N/A
SARS-CoV-2 Omicron BA.4/5 pseudovirus gene fragment	Integrated DNA Technologies	N/A
SARS-CoV-2 Omicron BQ.1.1 pseudovirus gene fragment	Integrated DNA Technologies	N/A
SARS-CoV-2 Omicron XBB.1 pseudovirus gene fragment	Integrated DNA Technologies	N/A
pPPI4 plasmid	Brouwer et al.^6^	N/A
pCR3 SARS-CoV-2–S_Δ19_ expression plasmid	GenBank	ID: MT449663.1
pHIV-1_NL43_ ΔEnv-NanoLuc reporter virus plasmid	Schmidt et al., 2020	N/A
pFastBac vector	Ekiert et al.^56^	N/A

**Software and algorithms**		

Microsoft Excel	Microsoft	RRID:SCR_016137
GraphPad Prism v8.3.0	GraphPad	N/A
FlowJo X software	BD Biosciences	N/A
HKL2000	Otwinowski et al.^57^	N/A
Repertoire Builder	Schritt et al.^58^	N/A
PHASER	McCoy et al.^59^	N/A
COOT	Emsley et al.^60^	N/A
PHENIX	Adams et al.^61^	N/A
Leginon automated image collection software	Suloway et al.^62^	N/A
DogPicker	Voss et al.^63^	N/A
Relion 3.0	Scheres et al.^64^	N/A

**Other**		

Vivaspin 20, 10.000 kDa MWCO, Polyethersulfone	Sartorius	Cat# VS.2001
Vivaspin 20, 50.000 kDa MWCO, Polyethersulfone	Sartorius	Cat# VS.2032
Vivaspin 20, 100.000 kDa MWCO, Polyethersulfone	Sartorius	Cat# VS.2041
Ni-NTA agarose beads	QIAGEN	Cat# 30210
Ni-NTA HighSorb plates	QIAGEN	Cat# 35061
Octet K2 system	ForteBio	N/A
Octet Biosensors: NiNTA	ForteBio	Cat# 18-5101
ARIA-SORP-II 4 lasers	BD Biosciences	N/A
FACS Canto II analyser	BD Biosciences	N/A
GloMax system	Turner BioSystems	Cat# 9101-002
Glutamax supplement	Thermo Fisher Scientific	Cat# 35050061
CrystalMation system	Rigaku	N/A
Synchrotron Radiation Lightsource (SSRL)	Stanford	N/A
FreeStyle 293 Expression medium	Gibco	Cat# 12338001
Dulbecco’s Modified Eagle Medium (DMEM)	Gibco	Cat# 11966025
Opti-MEM I reduced serum media	Gibco	Cat# 15392402
Protein G Agarose	Thermo Scientific Pierce	Cat# 10016363
NanoDrop One	Thermofisher	Cat# ND-ONE-W
SteriTop Filter Units	Merck Millipore	Cat# C3239
Streptavidin AF647	Biolegend	Cat# 405237
Streptavidin BV421	Biolegend	Cat# 405226
Crystal structure of SARS-CoV-2 receptor binding domain in complex with human antibody COVA309-22	This paper	PDB: 8F0I

### Resource availability

#### Lead contact

Further information and requests for resources and reagents should be directed to and will be fulfilled by the lead contact, Rogier W. Sanders (r.w.sanders@amsterdamumc.nl).

#### Materials availability

The reagents generated in this study can be retrieved upon reasonable request to the lead contact.

### Experimental model and study participant details

#### Patient sample

Blood from a SARS-CoV-2 Gamma-infected adult (COSCA309) was collected approximately 40 days after symptom onset through the COVID-19-specific antibodies (COSCA) study (NL 73281.018.20). The participant had a SARS-CoV-2 positive nasopharyngeal swab, as assessed by qRT-PCR (Roche LightCycler480, targeting the Envelope-gene 113bp). After density gradient centrifugation, peripheral blood mononuclear cells (PBMCs) of the donor were isolated. The COSCA study was conducted at the Amsterdam University Medical Centre, location AMC, the Netherlands and approved by the local ethical committee. Written informed consent was provided by the participant before being enrolled in the study.

### Method details

#### Protein constructs design and production

The mutations present in the stabilized S proteins compared to the WT strain (Wuhan Hu-1; GenBank: MN908947.3) are reported in Table S3. Soluble RBD protein constructs of the different variants were designed with the same mutations reported in the table (residues 319-541). gBlock gene fragments (Integrated DNA Technologies) of the different constructs were ordered and cloned into a pPPI4 avidin-tagged and/or hexahistidine-tagged vector by PstI-BamHI digestion and ligation with Gibson Assembly (Thermo Fisher Scientific). The recombinant human ACE-2 receptor was obtained in the same way after ordering the corresponding gBlock gene fragment (Integrated DNA Technologies). Sanger sequencing was used to verify the constructs. Proteins were then produced in human embryonic kidney (HEK)293F cells (Thermo Fisher Scientific) maintained in Freestyle medium (Life Technologies). Briefly, HEK293F cells were transfected with a 1:3 ratio of expression DNA plasmids (312.5 μg/l) and Polyethylenimine Hydrochloride (PEI)max (1 μg/μl) in OptiMEM. Supernatants containing the produced proteins were harvested six days after transfection, centrifuged at 4000 rpm for 30 min and filtered using 0.22 μM Steritop filter units (Merck Millipore). Affinity chromatography with Ni-NTA agarose beads (Qiagen) was used to purify the proteins. Eluates were subsequently concentrated and buffer exchanged to phosphate-buffered saline (PBS) or TN75 buffer (75 mM NaCl and 20 mM Tris HCl, pH 8.0) using VivaSpin20 filters (Sartorius). Protein concentrations were determined by the Nanodrop.

#### Probe staining and single B cell sorting

To produce fluorescent labelled-probes for the B cell sorting, soluble avidin-tagged Gamma S protein was biotinylated with a BIrA500 biotin-ligase reaction kit according to the manufacturer’s instruction (Avidity). Biotinylated protein was then mixed with streptavidin fluorophores (AF647, BioLegend; BV421, BioLegend), as described previously,^6^ and incubated at 4°C for 1 h. 10mM biotin (Genecopoiea) was added for at least 10 min to quench unbound streptavidin conjugates. Labelled proteins were then mixed with PBMCs for 30 min at 4°C, washed with FACS buffer (PBS supplemented with 1 mM EDTA and 2% fetal calf serum) and stained with a live/dead marker (viability-eF780, eBiosciences) together with CD19-AF700 (HIB19, BioLegend), CD20-PE-CF594 (2H7, BD Biosciences), CD27-PE (L128, BD Biosciences), IgM-BV605 (MHM-88, BioLegend), IgG-PE-Cy7 (G18-145, BD Biosciences). Sample was washed twice and acquired on the ARIA-SORP-II 4 lasers for B cell sorting. The lymphocyte population was first gated based on the morphology (FSC-A and SSC-A) and doublets were removed. Live B cells (CD19^+^Via^-^) double positive for the SARS-CoV-2 Gamma S protein (AF647 and BV421) were single cell-sorted into 96-well plates containing lysis buffer (20 U RNAse inhibitor (Invitrogen), first strand SuperScript III buffer (Invitrogen), and 1.25 μl of 0.1 M DTT (Invitrogen)). After single cell sorting, the plates were stored at -80°C for at least 1 h before a reverse transcriptase (RT)-PCR was performed to convert the mRNA of the lysed B cells into cDNA, as described previously.^6^ Analysis was performed using FlowJo X software (BD Biosciences).

#### Single-cell immunoglobulin gene amplification, cloning and antibodies expression

After RT-PCR, additional PCR rounds were performed to amplify the V(D)J variable region of the HC and LC of the antibodies, as described previously.^6^ PCR products were then cloned into corresponding human IgG1 expression vectors with Gibson Assembly (Thermo Fisher Scientific) and the mixture was subsequently transformed into DH5α cells. After DNA purification, the sequences were verified by Sanger sequencing. For small scale expression of the antibodies, adherent HEK293T cells (ATCC, CRL-11268) were cultured in Dulbecco’s Modified Eagle Medium (DMEM) supplemented with 10% foetal calf serum and a mixture of penicillin/streptomycin (100 U/ml and 100 μg/ml, respectively). HEK293T cells were seeded in 24-well plates at a density of 2.75x10^5^ cells/well 24 h prior to transfection. The transfection was performed with a 1:1 (w:w) HC/LC ratio using a 1:2.5 ratio with 1 mg/l PEImax (Polysciences) in 200 μl Opti-MEM. The transfection mix was incubated for 15 min at RT and then added onto the cells. Supernatants of transfected cells were harvested 48 h post-transfection and tested for preliminary screening by flow cytometry.

#### Larger expression of antibodies in HEK293F cells

Suspension HEK293F cells (Invitrogen) were maintained in FreeStyle medium (Gibco) and co-transfected with a 1:3 ratio of the two HC/LC DNA plasmids and 1 mg/l PEImax (Polysciences). Five days post-transfection, the cell suspension was centrifuged at 4000 rpm for 30 min, followed by filtration of the supernatant using 0.22 μm pore size SteriTop filters (Millipore). The filtered supernatant containing the recombinant IgG antibodies was run over a 10 ml protein G column (Pierce) and the antibodies were then eluted with 0.1 M glycine pH 2.5, into the neutralization buffer (1 M TRIS pH 8.7). 50 kDa VivaSpin20 columns (Sartorius) were used to concentrate and buffer exchange the antibodies to PBS. The IgG concentration was determined by the NanoDrop.

#### Flow cytometry-based screening of supernatants and purified recombinant mAbs

Cell surface-expressed SARS-CoV-2 ancestral and variant S were obtained by transfecting 8 μg of SARS-CoV-2 full-length DNA plasmid with 25 μl PEImax in 400 μl OptiMEM onto 12 to 15 ml HEK293T cells in a petri dish (seeded the day before at a density of 3.0x10^6^). After 48 h, cells were harvested and frozen until further use. After thawing, HEK293T cells expressing the S of interest were seeded at 20.000 to 30.000 cells per 96-well in FACS buffer (PBS/0.5% FCS) and were incubated 1:1 with non-purified supernatants from small scale IgG production or a dilution of purified HEK293F-produced mAbs for 30 min at 4°C. Subsequently, cells were washed twice with FACS buffer and incubated for 30 min at 4°C with 1:1000 diluted PE-conjugated goat F(ab)’2 anti-human IgG (Southern Biotech 2042-09) in the dark. Cells were washed and analysed on the FACS Canto II analyser. Samples were analysed by FlowJo X software (BD Biosciences) and percentage of cells that showed binding were plotted correspondingly.

#### Pseudovirus design and neutralization assay

The WT D614G, Alpha, Beta, Gamma, Delta, Omicron BA.1, BA.2, BA.4/5, BQ.1.1 and XBB.1 pseudovirus constructs were ordered as gBlock gene fragments (Integrated DNA Technologies) and cloned using Gibson Assembly (ThermoFisher), as described previously.^6^ Briefly, all S constructs were produced by co-transfecting HEK293T cells with the plasmid expressing the S with the pHIV-1NL43 ΔEnv-NanoLuc reporter virus plasmid. Cell supernatant containing the pseudovirus was harvested 48 hours post-transfection and stored at -80°C until further use. To test the neutralization activity, mAbs, bsAbs and antibody cocktails were serially diluted in 3-fold steps starting at a concentration of 10, 2.7 and 5.4 μg/ml, respectively. They were then mixed with the pseudovirus and incubated for 1 h at 37°C. The mixes containing the pseudovirus and the mAbs/bsAbs were then added to HEK293T-ACE2 cells, seeded the day before in poly-L-lysine-coated 96-well plates at a density of 20.000 cells/well. After 48 h, the pseudovirus-mAbs/bsAbs combination was removed, cells were lysed and transferred to half-area 96-wells white microplates (Greiner Bio-One). Luciferase activity of cell lysate was measured using the Nano-Glo Luciferase Assay System (Promega) with a Glomax plate reader (Turner BioSystems).

#### Authentic virus neutralization assay

We tested mAbs and bsAbs for their neutralization capacity against the ancestral SARS-CoV-2 virus (German isolate; GISAID ID EPI_ISL 406862; European Virus Archive Global #026V-03883) and VOCs, as previously described.^65^ Briefly, samples were serially diluted in Dulbecco modified Eagle medium supplemented with NaHCO_3_, HEPES buffer, penicillin, streptomycin, and 1% fetal bovine serum, starting at a dilution of 10 μg/mL in 50 μl. Subsequently, 50 μL of virus suspension were added to each well and incubated at 35°C for 1 h. Vero E6 cells were added in a concentration of 20.000 cells per well and subsequently incubated for 48 hours at 35°C. After incubation, cells were fixed with 4% formaldehyde/phosphate-buffered saline (PBS) and stained with a nucleocapsid targeting monoclonal antibody. Bound ab as a measure for infected cells was detected using horseradish peroxidase–conjugated goat anti-human IgG (1:3000) in 2% milk/PBS for 1 hour at RT. After washing, the color reaction was developed using 3,3, 5,5’-tetramethylbenzidine substrate (Thermo Scientific Scientific). The reaction was stopped by adding 0.8 N sulfuric acid, and OD450 (optical density at 450 nm) was measured using standard equipment.

#### Ni-NTA enzyme-linked immunosorbent assay

Soluble hexahistidine-tagged SARS-CoV-2 WT, Alpha, Beta, Gamma, Delta, Omicron BA.1, BA.2, BA.4/5, BQ.1.1 and XBB.1 RBD proteins (1μg/ml) were loaded in casein (Thermo Scientific) on 96-well Ni-NTA plates (Qiagen) for 2h at RT. After washing the plates with Tris-Buffered Saline (TBS), five-fold serial dilutions of mAbs (starting from 5 μg/ml) in casein were added to the plates. After incubating the plates for 2 h at RT, they were washed three times with TBS, and a 1:3000 dilution of HRP-labelled goat anti-human IgG (Jackson Immunoresearch) in casein was added for 1 h at RT. Plates were finally washed five times with TBS/20% Tween. Colorimetric detection was performed after developing the reaction for 4 min before termination by adding 0.8 M sulfuric acid.

#### Competition assays by biolayer interferometry

For competition experiments, an Octet K2 (ForteBio) was used. Briefly, to test the competition of the ACE-2 receptor with COVA309 mAbs, autologous hexahistidine-tagged Gamma S (10 μg/ml) was loaded in running buffer (PBS, 0.02% Tween20, 0.1% BSA) on Ni-NTA biosensors. To measure association, the chip was first dipped in running buffer to remove protein in excess, and subsequently dipped for 300s in a well containing 10 μg/ml mAb in running buffer. Next, the chip was dipped for 300s in 5 μg/ml of recombinant human ACE-2 in running buffer to measure competition with the mAbs.

For mAbs cross-competition, Ni-NTA biosensors were loaded with 10 μg/ml hexahistidine-tagged SARS-CoV-2 WT S protein in running buffer. After dipping the chip in running buffer to remove excess of S, the chip was transferred for 300 s to a well containing 10 μg/ml of one of the COVA309 mAbs in running buffer. Next, the chip was dipped for 300 s in either 10 μg/ml of previously characterized COVA mAbs^6^ and CR3022,^46^ or 5 μg/ml of J08 and S309 mAbs in running buffer to measure cross-competition.

#### Bispecific antibodies production

BsAbs were generated by introducing amino acid mutations (F405L or K409R) in the HC constant region of the parental mAbs by Q5 Site-Directed Mutagenesis (New England Biolabs). Individual mutated mAbs were produced in HEK293F cells and purified as reported above. BsAb combinations were generated by using a previously described controlled Fab-arm exchange protocol^66^ and later tested for neutralization against SARS-CoV-2 VOC pseudo and primary viruses.

#### Expression and purification of antibody fragments (Fabs)

The plasmids were transiently co-transfected into ExpiCHO cells at a ratio of 2:1 (HC:LC) using ExpiFectamine™ CHO Reagent (Thermo Fisher Scientific), according to the manufacturer’s instructions. The supernatant was collected at 10 days post-transfection. The Fabs were purified with a CaptureSelect™ CH1-XL Affinity Matrix (Thermo Fisher Scientific), followed by size exclusion chromatography.

#### Expression and purification of SARS-CoV-2 RBD for crystallization

Expression and purification of the SARS-CoV-2 RBD for crystallization were performed as described previously.^46^ Briefly, the RBD (residues 333-529) of the SARS-CoV-2 S protein (GenBank: QHD43416.1) was cloned into a customized pFastBac vector^56^ and fused with an N-terminal gp67 signal peptide and C-terminal His_6_ tag.^46^ A recombinant bacmid DNA was generated using the Bac-to-Bac system (Life Technologies). Baculovirus was generated by transfecting purified bacmid DNA into Sf9 cells using FuGENE HD (Promega), and subsequently used to infect suspension cultures of High Five cells (Life Technologies) at an MOI of 5 to 10. Infected High Five cells were incubated at 28°C with shaking at 110 rpm for 72 h for protein expression. The supernatant was then concentrated using a 10 kDa MW cutoff Centramate cassette (Pall Corporation). The RBD protein was purified by Ni-NTA, followed by size exclusion chromatography, and buffer exchanged into 20 mM Tris-HCl pH 7.4 and 150 mM NaCl.

#### Crystallization and structural determination

The COVA309-22/RBD complex was formed by mixing each of the protein components at an equimolar ratio and incubating overnight at 4°C. The protein complex was adjusted to 11 mg/ml and screened for crystallization using the 384 conditions of the JCSG Core Suite (Qiagen) on our robotic CrystalMation system (Rigaku) at Scripps Research. Crystallization trials were set-up by the vapor diffusion method in sitting drops containing 0.1 μl of protein and 0.1 μl of reservoir solution. Optimized crystals were then grown in drops containing 0.1 M sodium cacodylate, pH 6.5, 0.2 M magnesium chloride, and 20% (w/v) polyethylene glycol 1000 at 20°C. Crystals appeared on day 3, were harvested on day 15 by soaking in reservoir solution supplemented with 20% (v/v) ethylene glycol, and then flash cooled and stored in liquid nitrogen until data collection. Diffraction data were collected at cryogenic temperature (100 K) at the Stanford Synchrotron Radiation Lightsource (SSRL) on Scripps/Stanford beamline 12-2. Diffraction data were processed with HKL2000.^57^ The model of COVA309-22 was generated by Repertoire Builder.^58^ Structures were solved by molecular replacement using PHASER.^59^ Models for molecular replacement were derived from PBD 6W41. Iterative model building and refinement were carried out in COOT^60^ and PHENIX,^61^ respectively. Epitope and paratope residues, as well as their interactions, were identified by accessing PISA at the European Bioinformatics Institute.^67^^,^^68^

#### Negative stain electron microscopy analysis

SARS-CoV-2 S protein was complexed with three-fold molar excess of Fab and incubated at RT for 30 min. The complex was diluted to 0.02 mg/ml in 1xTBS and 3 μl applied to a 400mesh Cu grid, blotted with filter paper, and stained with 2% uranyl formate. Micrographs were collected on a ThermoFisher Tecnai Spirit microscope operating at 120kV with a FEI Eagle CCD (4k) camera at 52,000 magnification using Leginon automated image collection software.^62^ Particles were picked using DogPicker^63^ and 3D classification was done using Relion 3.0.^64^

### Quantification and statistical analysis

Data visualization and statistical analysis were performed in GraphPad Prism Software (version 8.3).

## Data Availability

• All data reported in this paper will be shared by the lead contact upon request. • This paper does not report original code. • Any additional information required to reanalyse the data reported in this paper is available from the lead contact upon request. • This paper includes a new deposited crystal structure of the COVA309-22 antibody. The new data can be accessed from PDB https://doi.org/10.2210/pdb8F0I/pdb.
