# Supplementary material for: Broad SARS-CoV-2 neutralization by monoclonal and bispecific antibodies derived from a Gamma-infected individual
Source: iScience. 2023 Sep 22;26(10):108009. doi: 10.1016/j.isci.2023.108009 (PMC10570122; doi:10.1016/j.isci.2023.108009)
Supplement: Document S1. Figures S1 and S2 and Tables S1–S3 [file mmc1.pdf]

iScience, Volume *26*

Supplemental information

Broad SARS-CoV-2 neutralization by monoclonal and bispecific antibodies derived from a Gamma-infected individual

Denise Guerra, Tim Beaumont, Laura Radić, Gius Kerster, Karlijn van der Straten, Meng Yuan, Jonathan L. Torres, Wen-Hsin Lee, Hejun Liu, Meliawati Poniman, Ilja Bontjer, Judith A. Burger, Mathieu Claireaux, Tom G. Caniels, Jonne L. Snitselaar, Tom P.L. Bijl, Sabine Kruijer, Gabriel Ozorowski, David Gideonse, Kwinten Sliepen, Andrew B. Ward, Dirk Eggink, Godelieve J. de Bree, Ian A. Wilson, Rogier W. Sanders, and Marit J. van Gils

This PDF file includes:

Supplementary Figures 1 and 2

Supplementary Tables 1, 2 and 3

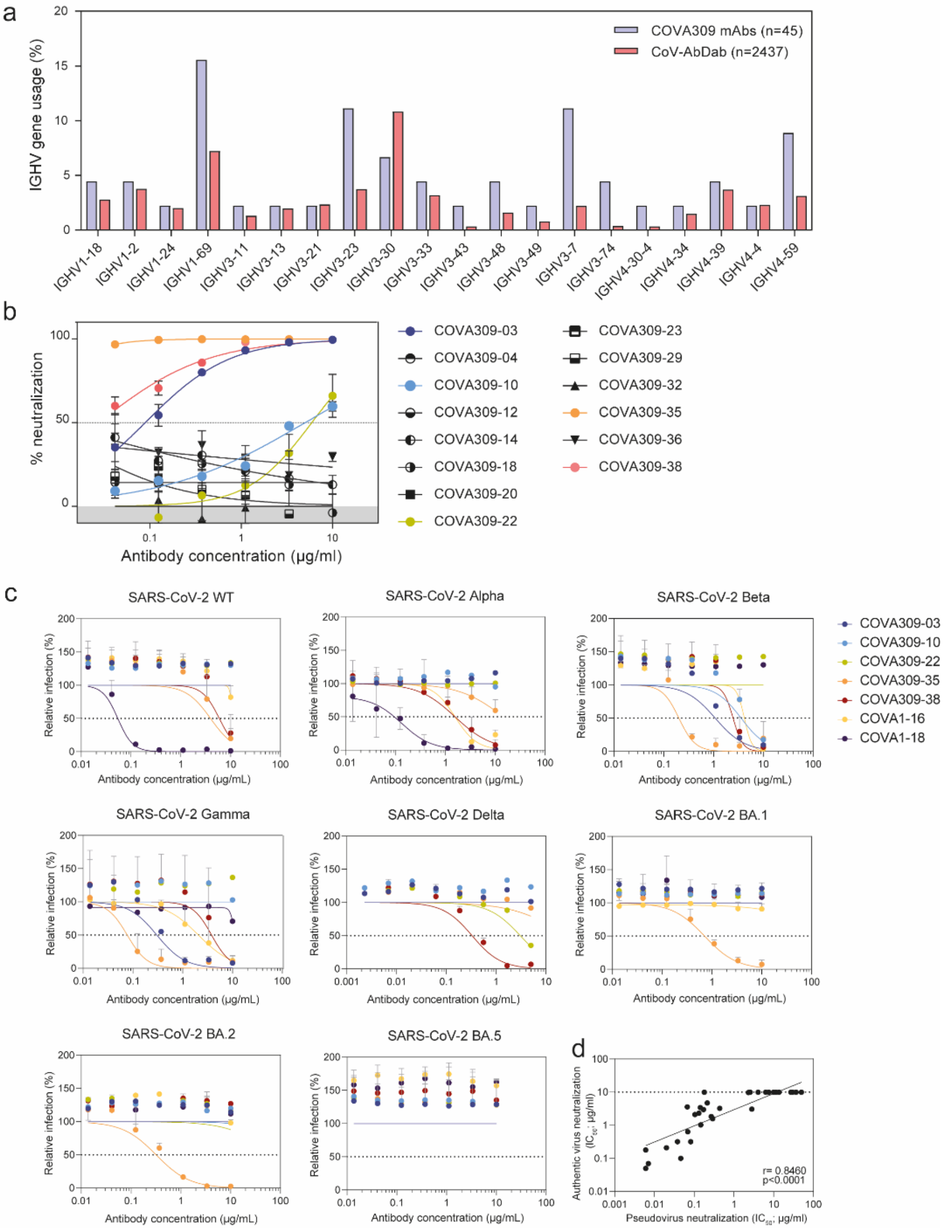

Supplementary Figure 1. Characterization of the COVA309 mAbs, related to Figure 1.

a. Bar graph showing the mean IGHV gene usage (%) by the 293T-produced COVA309 mAbs (purple, n=45) versus sequences derived from WT-elicited B cells included in the CoVAbDab database[31] (red, n=2437). **b.** Percentage of neutralization of SARS-CoV-2 Gamma pseudovirus by the 14 HEK293F-produced COVA309 mAbs. COVA309-03, -10, -22, -35 and -38 showed considerable neutralizing activity against the autologous variant and were selected for further analysis. **c.** Full authentic virus neutralization curves for COVA309 mAbs, COVA1-16 and COVA1-18, which show small variation between repeats and strong

reproducibility of the data. **d.** Spearman correlation between data obtained in pseudovirus and authentic virus neutralization assays (IC_{50}; µg/ml). Dotted line indicates the IC_{50} cut-off for the authentic virus neutralization (10 µg/ml).

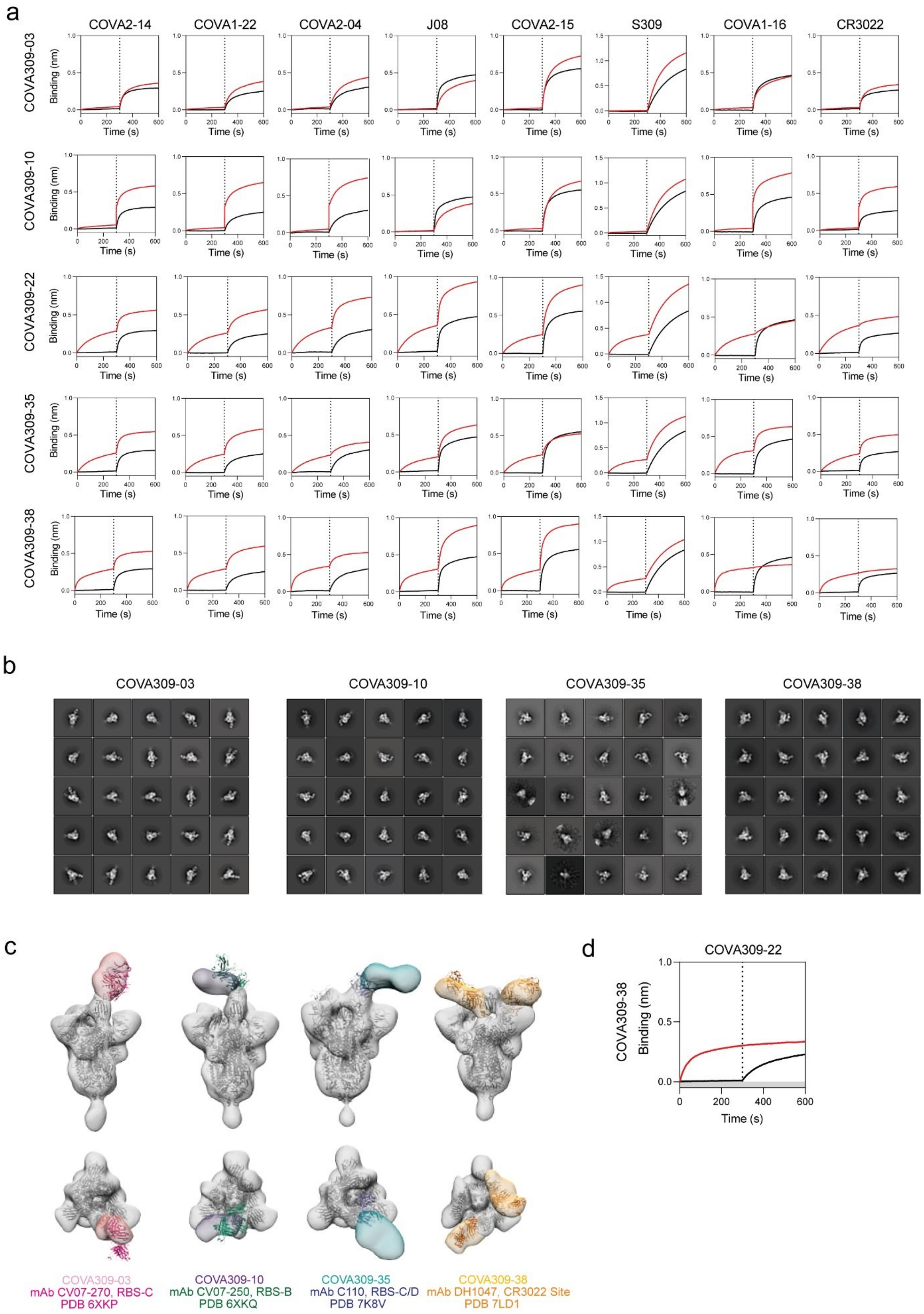
a
COVA2-14
COVA1-22
COVA2-04
J08
COVA2-15
S309
COVA1-16
CR3022
COVA309-03
COVA309-10
COVA309-22
COVA309-35
COVA309-38
Binding (nm)
Time (s)
b
COVA309-03
COVA309-10
COVA309-35
COVA309-38
c
COVA309-03
mAb CV07-270, RBS-C
PDB 6XKP
COVA309-10
mAb CV07-250, RBS-B
PDB 6XKQ
COVA309-35
mAb C110, RBS-C/D
PDB 7K8V
COVA309-38
mAb DH1047, CR3022 Site
PDB 7LD1
d
COVA309-22
COVA309-38
Binding (nm)
Time (s)

Supplementary Figure 2. Epitope analysis of the COVA309 mAbs, related to Figure 2.

a. COVA309 mAbs competition to known SARS-CoV-2 mAbs, including COVA2-14 (S2), COVA1-22 (NTD), COVA2-04 (RBS-A), J08 (RBS-B), COVA2-15 (RBS-D), S309 (S309 site), COVA1-16 (CR3022 site) and CR3022 (CR3022 site)[6,43-45], as assessed by BLI. Black curves represent the baseline binding of the competitors, while the red curves show the binding of the competitors in the presence of the analytes. **b.** Representative 2D images from NS-EM analysis of Omicron 6P and Gamma 6P S trimers complexed with the COVA309-03, -10, -35 and -38 Fabs. **c.** Reference mAb models fitted into COVA309 mAbs density for RBS class assignment. mAbs C110 (blue; PDB:7K8V), CV07-250 (green; PDB: 6XKQ), CV07-270 (pink; PDB: 6XKP), DH1047 (orange; PDB: 7LD1)[46-47]. **d.** Cross-competition of COVA309-38 and COVA309-22 for binding to SARS-CoV-2 WT S, as measured by BLI assay. Red curve indicates competition between the mAbs, while the black curve corresponds to COVA309-22 S binding in the absence of the analyte.

Supplementary Table 1. Genetic signatures of COVA309 mAbs, related to Figure 1.

Table reporting the gene usage, V region identity (%) and CDR3 sequence for the produced Gamma-specific B cells. VH and VL region identity refers to V gene segment identity compared to the germline repertoire (International Immunogenetics Information System)[27].

mAb ID	HC gene usage	V region identity (%)	CDRH3 sequence	LC gene usage	V region identity (%)	CDRL3 sequence
COVA309-01	IGHV1-2, IGHJ5, IGHD3-10	95.14	CVRGDYHGSGSYYWSWWFDPW	IGLV1-51, IGLJ2	97.89	CGTWDSSLSAGLF
COVA309-02	IGHV1-69, IGHJ6, IGHD5-24	94.10	CARGGWGKDGQIDNYYYYYMDVW	IGLV2-11, IGLJ1	95.83	CCSYAGNYIFVF
COVA309-03	IGHV3-48, IGHJ3, IGHD2-8	95.14	CARLLMGAFDIW	IGKV1-5, IGKJ2	97.49	CHQYNSYPYTF
COVA309-04	IGHV3-7, IGHJ6, IGHD4-11	98.26	CARDYSNYDLYMDVW	IGKV1-5, IGKJ1	96.77	CQQYSTYSRTF
COVA309-05	IGHV1-69, IGHJ3, IGHD2-2	96.88	CATESRGGYQLLSPHDAFDIW	IGKV4-1, IGKJ4	98.65	CQQYYSTPLTL
COVA309-06	IGHV3-49, IGHJ6, IGHD3-9	98.98	CTARGYYDILTGYYSGYMDVW	IGKV1-33, IGKJ2	95.70	CQQNDNLPYTF
COVA309-07	IGHV3-13, IGHJ6, IGHD3-16	97.89	CARGVTVRGFSLYYYYMDVW	IGKV1-39, IGKJ2	98.57	CQQSYSNPPEGSF
COVA309-08	IGHV4-30-4, IGHJ4, IGHD3-3	97.25	CARGPGPKDFWSGTALDYW	IGKV1-27, IGKJ4	99.28	CQKYNSALTF
COVA309-09	IGHV3-23, IGHJ3, IGHD3-22	100.00	CAKDPNYYDSSGLFAFDIW	IGKV1-6, IGKJ3	97.13	CLQDYNYPFTF
COVA309-10	IGHV3-23, IGHJ6, IGHD1-20	99.31	CAKASGPAATRITGTPAYYYYMDVW	IGKV1-39, IGKJ5	97.85	CQQSYSTPPTF
COVA309-11	IGHV4-59, IGHJ6, IGHD3-22	96.49	CATYYYDSSGYSYGMDVW	IGKV1-9, IGKJ4	97.49	CQQLNSYPLTF
COVA309-12	IGHV3-30, IGHJ6, IGHD5-12	98.61	CAKDRGYGNYYYGMDVW	IGKV1-6, IGKJ4	95.70	CLQDYTYPLTF
COVA309-13	IGHV1-69, IGHJ4, IGHD3-10	97.57	CARDPGDYYGSGSYSLLDYW	IGLV2-11, IGLJ1	97.57	CCSYAGTYTWVF

COVA309-14	IGHV3-7, IGHJ6, IGHD3-22	98.26	CARVVVLGYDTAYYYYYGLDVW	IGKV3-20, IGKJ3	98.23	CQQYGSSPPIFTF
COVA309-15	IGHV1-69, IGHJ4, IGHD3-10	97.92	CARVSGYSASGSYEDYW	IGKV1-33, IGKJ5	97.85	CQQYHNLPITF
COVA309-16	IGHV3-7, IGHJ4, IGHD3-22	98.26	CAREETAYYYDTGGQYIEYYFDYW	IGLV7-46, IGLJ3	94.79	CFLYYSGVRVL
COVA309-17	IGHV4-59, IGHJ5, IGHD6-13	97.54	CARHYVAAAGTYNWFDPW	IGKV1-33, IGKJ3	96.42	CHQYDNLR
COVA309-18	IGHV3-23, IGHJ4, IGHD2-15	98.26	CAKDGGRLPLYYFDYW	IGKV1-5, IGKJ1	97.85	CQQYNSYPWTF
COVA309-19	IGHV3-11, IGHJ4, IGHD3-9	99.65	CARAGGGMVLTGYYLYYFDYW	IGKV3-20, IGKJ4	98.58	CQQYGSSLLTF
COVA309-20	IGHV3-7, IGHJ6, IGHD6-19	98.26	CAGVGYRSGWYVVGGMDVW	IGKV1-5, IGKJ1	98.21	CQQYNTYSATF
COVA309-21	IGHV3-74, IGHJ3, IGHD1-26	93.75	CARESARYRLPEFW	IGLV9-49, IGLJ2	94.95	CGADHGTGSKFVYLF
COVA309-22	IGHV3-33, IGHJ4, IGHD4-17	98.96	CARDEGTMTTYFDYW	IGKV1-39, IGKJ1	97.13	CQQSYSTPPWTF
COVA309-23	IGHV3-23, IGHJ3, IGHD3-22	98.26	CAKDPNYYDSSGVFAFDIW	IGKV1-5, IGKJ2	97.49	CQQYNTYSYTF
COVA309-24	IGHV4-59, IGHJ4, IGHD4-23	98.95	CARHLADYGENGYFDYW	IGKV1-6, IGKJ5	97.13	CLQDYNYPITF
COVA309-25	IGHV3-21, IGHJ2, IGHD2-21	97.22	CARERAVTAGTPRYFDLW	IGKV1-33, IGKJ4	97.85	CQQYDNLPTF
COVA309-26	IGHV3-43D, IGHJ4, IGHD1-26	98.61	CAKDRVGILGATDGFDYW	IGKV1-9, IGKJ4	97.85	CQQLNSYPLTF
COVA309-27	IGHV1-24, IGHJ3, IGHD2-15	96.18	CATAVWGVLIAATGGFDFW	IGKV3-20, IGKJ4	96.45	CHQYGSSPLTF
COVA309-28	IGHV3-48, IGHJ3, IGHD3-16	98.61	CARSWVNDAFDIW	IGKV1-39, IGKJ1	98.92	CQQSYSTPWTF
COVA309-29	IGHV3-30, IGHJ5, IGHD6-19	98.26	CAKAVAGYNYFDPW	IGKV1-9, IGKJ3	97.49	CQQLKSYPLFTF
COVA309-30	IGHV4-39, IGHJ4, IGHD3-10	98.28	CARLLWLRGDFGYW	IGLV6-57, IGLJ3	99.31	CQSYDSSIWVF
COVA309-31	IGHV1-2, IGHJ5, IGHD3-10	98.26	CAREGITMVRGVRSWFDPW	IGLV2-23, IGLJ3	96.18	CCSYAGSSTWVF

COVA309-32	IGHV4-59, IGHJ2	97.89	CARGFDLW	IGKV3-20, IGKJ1	97.52	CQQYGSSPWTF
COVA309-33	IGHV4-4, IGHJ6, IGHD3-3	97.54	CAGGGFLEWLLYPHLNYMDVW	IGLV3-25, IGLJ2	84.59	CQSADSSGTVVF
COVA309-34	IGHV3-23, IGHJ4, IGHD2-2	95.49	CAKFLEDGVGFCSSPGCYTLDYW	IGKV1-12, IGKJ3	75.27	CQQANNFPFTF
COVA309-35	IGHV1-69, IGHJ6, IGHD2-21	98.26	CARDEGETTPLAYYYYYMDVW	IGKV1-27, IGKJ4	97.49	CQKYNSALALTF
COVA309-36	IGHV3-30, IGHJ4, IGHD5-24	97.92	CAKGRGGYDTYFDYW	IGKV1-5, IGKJ2	97.13	CQQYNSYVYTF
COVA309-37	IGHV4-39, IGHJ4, IGHD1-26	95.53	CASSFGSYYVAFDYW	IGLV2-11, IGLJ2	98.61	CCSYAGSYTLVVF
COVA309-38	IGHV3-7, IGHJ4, IGHD5-24	96.88	CAKSAWLQGDFDYW	IGLV6-57, IGLJ3	98.97	CQSYDTGNQVF
COVA309-39	IGHV1-69, IGHJ6, IGHD6-13	98.61	CARGGLRPAAMSYQYSSSWYRYMDVW	IGKV3-20, IGKJ1	98.23	CQQYGSSPSGTF
COVA309-40	IGHV1-18, IGHJ4, IGHD3-22	98.26	CARDDYFDSSGYYYAWDYW	IGLV1-47, IGLJ3	98.25	CAAWDDSLSGPVF
COVA309-41	IGHV1-18, IGHJ1, IGHD6-13	94.79	CVRGSSSTYGGVQHW	IGLV6-57, IGLJ3	95.88	CQSYDSTNQVF
COVA309-42	IGHV1-69, IGHJ4, IGHD3-10	99.31	CARVSGYSASGSYEDYW	IGLV1-44, IGLJ3	95.79	CASWDDGLSGWVF
COVA309-43	IGHV3-33, IGHJ4, IGHD5-18	97.92	CARDFRVSDYTFGNW	IGLV3-10, IGLJ3	96.77	CYSRDSSGNLWVF
COVA309-44	IGHV3-74, IGHJ4, IGHD3-9	94.10	CARDFTGPFDYW	IGLV1-47, IGLJ1	95.79	CVVWDDSLSGYVF
COVA309-45	IGHV4-34, IGHJ6, IGHD5-12	95.79	CASRQGVAEIYGMDVW	IGKV3-15, IGKJ4	97.85	CQQYNKWPPLTF

Supplementary Table 2. X-ray data collection and refinement statistics, related to Figure 2.

**Data collection**	COVA309-22 + SARS-CoV-2 RBD
Beamline	SSRL12-1
Wavelength (Å)	0.9795
Space group	$P\ 2_1\ 2_1\ 2_1$
Unit cell parameters a, b, c (Å)	54.7, 152.5, 240.8
α, β, γ (°)	90, 90, 90
Resolution (Å) [a]	50.0-3.70 (3.76-3.70)
Unique reflections [a]	22,407 (2,111)
Redundancy [a]	12.4 (9.5)
Completeness (%) [a]	99.8 (98.9)
$<I/\sigma_I>$ [a]	4.9 (0.9)
R_{sym}[b] (%) [a]	37.6 (>100)
R_{pim}[b] (%) [a]	11.1 (57.7)
$CC_{1/2}$[c] (%) [a]	99.6 (81.3)
**Refinement statistics**	
Resolution (Å)	46.8-3.70
Reflections (work)	22,382
Reflections (test)	2,111
R_{cryst}[d] / R_{free}[e] (%)	22.6/27.5
No. of copies in ASU	3
No. of atoms	14,610
Fab	9,939
RBD	4,671
Average *B*-values ($Å^2$)	81
Fab	82
RBD	81
Wilson *B*-value ($Å^2$)	87
**RMSD from ideal geometry**	
	0.002
Bond angle (°)	0.57
Bond length (Å)	
**Ramachandran statistics (%)**	
[f] Favored	92.2
Outliers	0.06
**PDB code**	8F0I

[a] Numbers in parentheses refer to the highest resolution shell.

[b] $R_{sym} = \Sigma_{hkl} \Sigma_i | I_{hkl,i} - <I_{hkl}> | / \Sigma_{hkl} \Sigma_i I_{hkl,i}$ and $R_{pim} = \Sigma_{hkl} (1/(n-1))^{1/2} \Sigma_i | I_{hkl,i} - <I_{hkl}> | / \Sigma_{hkl} \Sigma_i I_{hkl,i}$, where $I_{hkl,i}$ is the scaled intensity of the ith measurement of reflection h, k, l, $<I_{hkl}>$ is the average intensity for that reflection, and *n* is the redundancy. [c] $CC_{1/2}$ = Pearson correlation coefficient between two random half datasets. [d] $R_{cryst} = \Sigma_{hkl} | F_o - F_c | / \Sigma_{hkl} | F_o |$ x 100, where F_o and F_c are the observed and calculated structure factors, respectively. [e] R_{free} was calculated as for R_{cryst}, but on a test set comprising 5% of the data excluded from refinement. [f] From MolProbity[67].

Supplementary Table 3. Mutations present in the S protein constructs and pseudoviruses compared to the WT strain, related to Figure 1 and 3.

Alpha	Beta	Gamma	Delta	Omicron BA.1	Omicron BA.2	Omicron BA.4/5	Omicron BQ.1.1	Omicron XBB.1
deletion (Δ) of H69-V70<br>ΔY144<br>N501Y<br>A570D<br>D614G<br>P681H<br>T716I<br>S982A<br>D1118H	L18F<br>D80A<br>D215G<br>L242H<br>R246I<br>K417N<br>E484K<br>N501Y<br>D614G<br>A701V	L18F<br>T20N<br>P26S<br>D138Y<br>R190S<br>K417T<br>E484K<br>N501Y<br>D614G<br>H655Y<br>T1027I	T19R<br>G142D<br>E156G<br>Δ157-158<br>L452R<br>T478K<br>D614G<br>P681R<br>D950N	A67V<br>Δ69-70<br>T95I<br>G142D<br>Δ143-145<br>Δ211<br>L212I<br>ins214EPE<br>G339D<br>S371L<br>S373P<br>S375F<br>K417N<br>N440K<br>G446S<br>S477N<br>T478K<br>E484A<br>Q493K<br>G496S<br>Q498R<br>N501Y<br>Y505H<br>T547K<br>D614G<br>H655Y<br>N679K<br>P681H<br>N764K<br>D796Y<br>N856K<br>Q954H<br>N969K<br>L981F	T19I<br>Δ24-26<br>A27S<br>G142D<br>V213G<br>G339D<br>S371F<br>S373P<br>S375F<br>T376A<br>D405N<br>R408S<br>K417N<br>N440K<br>S477N<br>T478K<br>E484A<br>Q493R<br>Q498R<br>N501Y<br>Y505H<br>D614G<br>H655Y<br>N679K<br>P681H<br>N764K<br>D796Y<br>Q954H<br>N969K	T19I<br>L24S<br>Δ25-27<br>Δ69-70<br>G142D<br>V213G<br>G339D<br>S371F<br>S373P<br>S375F<br>T376A<br>D405N<br>R408S<br>K417N<br>N440K<br>L452R<br>S477N<br>T478K<br>E484A<br>F486V<br>Q498R<br>N501Y<br>Y505H<br>D614G<br>H655Y<br>N679K<br>P681H<br>N764K<br>D796Y<br>Q954H<br>N969K	T19I<br>Δ24-26<br>A27S<br>Δ69-70<br>G142D<br>V213G<br>G339D<br>R346T<br>S371F<br>S373P<br>S375F<br>T376A<br>D405N<br>R408S<br>K417N<br>N440K<br>K444T<br>L452R<br>N460K<br>S477N<br>T478K<br>E484A<br>F486V<br>Q498R<br>N501Y<br>Y505H<br>D614G<br>H655Y<br>N679K<br>P681H<br>N764K<br>D796Y<br>Q954H<br>N969K	T19I<br>Δ24-26<br>A27S<br>V83A<br>G142D<br>Y145Q<br>Δ146<br>Q183E<br>V213E<br>G252V<br>G339H<br>R346T<br>L368I<br>S371F<br>S373P<br>S375F<br>T376A<br>D405N<br>R408S<br>K417N<br>N440K<br>V445P<br>G446S<br>N460K<br>S477N<br>T478K<br>E484A<br>F486S<br>F490S<br>Q498R<br>N501Y<br>Y505H<br>D614G<br>H655Y<br>N679K<br>P681H<br>N764K<br>D796Y<br>Q954H<br>N969K
